# Regulation of the mitochondrial transition pore: impact on mammalian aging

**DOI:** 10.18632/aging.100259

**Published:** 2011-01-16

**Authors:** Heinz D. Osiewacz

**Affiliations:** Johann Wolfgang Goethe University, Faculty for Biosciences & Cluster of Excellence Molecular Complexes, Institute of Molecular Biosciences, 60438 Frankfurt, Germany

During the last few years, data from different biological systems have revealed evidence for an age-related increase in the abundance of CypD, a peptidyl prolylcis, trans-isomerase located in the mitochondrial matrix. Such an increase was reported in the short-lived aging model organism *Podospora anserina* [1,2] but also in rat gastrocnemius muscle and in brain tissue of mice and humans [2-3]. The enzyme is known to be involved in the regulation of the mitochondrial transition pore (mPTP) [4], a macromolecular mitochondrial protein complex the structure of which is still unsolved and controversial. The activity of CypD can be repressed by the cyclosporine A, a cyclic peptide used in human transplantation medicine or in treatment of Ullrich congenital dystrophy. In a recent aging study, a strong effect of CypD overexpression on mitochondrial ultrastucture structure and on aging was reported in *P. anserina* [5]. Overexpression strains were characterized by mitochondrial dysfunction and accelerated aging as the result of the induction of apoptosis. Treatment of CypD overexpressors was found to be effective in a reversion of the accelerated aging phenotype and it was suggested that cyclosporine A or other molecules affecting the function of CypD may be promising compounds for the development of anti-aging interventions by interfering into mitochondrial apoptotis pathways. In *P. anserina* apoptosis is the final pathway leading to death of this simple microbial aging model [6-9]. In contrast, in mammalian systems, it is rather represents a pathway relevant for development and protection against cancer. However, more and more data accumulate which also point to an important role of apoptosis in the final life stage of mammals. For exemple, transgenic mice expressing a mutated gene of the mitochondrial DNA polymerase gamma, which, due to loss of the proof reading activity of this enzyme, express an accelerated aging phenotype with sarcopenia and an induction of apoptosis markers [10,11].

In the last 2010 issue of AGING [12] Hafner et al. report the regulation of the mPTP of mice via deacetylation of CypD at lysine 166 by the NAD^+^-dependent SIRT3 deacetylase. Mice lacking SIRT3 display an accelerated aging phenotype correlated with increased mitochondrial swelling as the result of mPTP opening. Most remarkably, this phenotype is related to heart impairments and can be reverted by treatment with cyclosporine A. Sirt3 knockout mice are hypersensitive to heart stress, display cardiac hypertrophy and fibrosis. From their work the authors suggest that the phenotype of the mice is the result of an initial reduction of SIRT3 activity leading to increases in susceptibility of mPTP formation. Subsequently this leads to impairments in NAD^+^ generation by mitochondria and, via a positive feedback cycle, to accelerated mitochondrial depolarization and destruction. In mammals, CypD thus appears to of relevance not only for skeletal muscle function but also in the heart connecting this pathway to one of the most important age-related death in humans, cardiac failure. Overall the data are an important piece of work towards the elucidation of the mechanistic details of mPTP regulation. Moreover, the work provides additional and very promising perspectives for the development of medical strategies to intervene into the part of the molecular network governing aging via mitochondrial pathways involved in the control of apoptosis as they have been put forward earlier [7,13].
